# Introducing the new CONSORT extension for stepped-wedge cluster randomised trials

**DOI:** 10.1186/s13063-018-3116-3

**Published:** 2019-01-18

**Authors:** Karla Hemming, Monica Taljaard, Jeremy Grimshaw

**Affiliations:** 1 Institute of Applied Health Research, University of Birmingham, Birmingham, B15 2TT UK; 2 Clinical Epidemiology Program, Ottawa Hospital Research Institute, 1053 Carling Avenue, Ottawa, ON K1Y4E9 Canada; 3 School of Epidemiology and Public Health, University of Ottawa, Ottawa, ON Canada; 4 Department of Medicine, University of Ottawa, Ottawa, ON Canada

## Abstract

The use of the stepped-wedge cluster randomised trial (SW-CRT) is on the increase, and although there are still relatively few SW-CRTs currently published its use is bound to show an increase in the near future. An extension of the CONSORT reporting guideline for SW-CRTs has recently been developed. By making reporting guidelines for this innovative design available relatively early in its development, it is possible that the methodological conduct and reporting of future SW-CRTs will not be at the same risk of low-quality of reporting as is the case with many other study designs. We provide a brief overview of this reporting guideline and encourage authors to use it appropriately; and for journal editors to endorse its use.

## What are stepped-wedge cluster randomised trials and why are they useful?

The SW-CRT is a novel type of cluster randomised trial that is increasingly being used, especially to evaluate health service delivery interventions or other cluster-level interventions [7]. The design is characterised by the fact that clusters are randomised to one of several different sequences which dictate the time at which the cluster will switch from the control condition to the intervention condition. The fundamental appeal of the study design is the fact that all clusters ultimately receive the intervention condition. This guarantee of receiving the intervention can increase the social appeal of the study and allows for an evaluation within the context of a routine roll-out. The design is typically used to evaluate how interventions would work in real-world settings, with limited exclusion criteria and are pragmatic rather than explanatory.

Whilst, to date, only about 40 completed SW-CRTs have been published, there has been an exponential increase in the use of this design over the past few years with an expected increase in the near future: there are currently around 80 published protocols. There are multiple other indicators of the upward trajectory of the design, included nationally funded methodological grants; dedicated conferences to the design; highly cited methodological papers and over 100 stepped-wedge trials listed on the three main trial registries as ‘ongoing’.

## Illustrative example of the SW-CRT

**Fig. 1:**
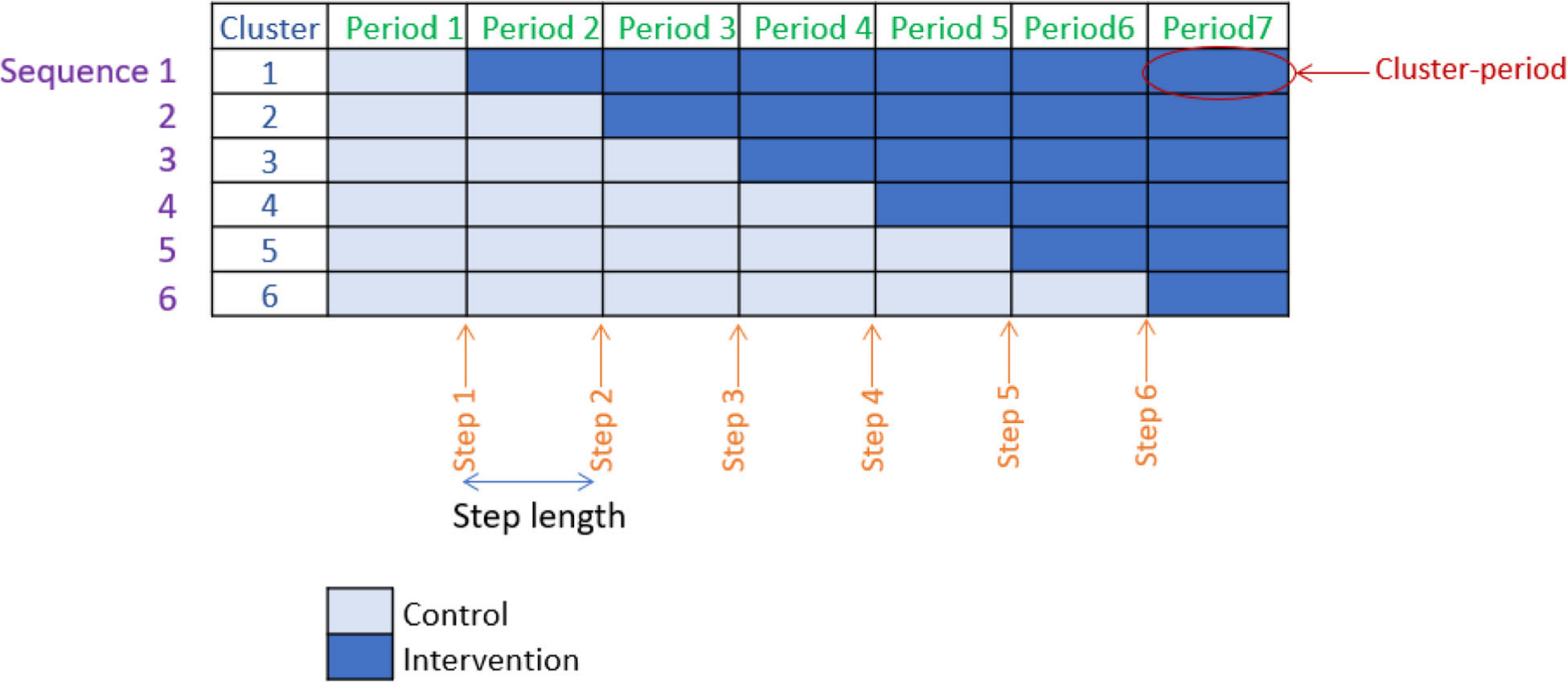
Example study diagram for the RegisterNow-1 stepped-wedge randomised controlled trial (SW-CRT) with key terminology as recommended by the Consolidated Standards of Reporting Trials (CONSORT) extension for SW-CRTs

Figure 1 presents an example study diagram for the most basic version of the SW-CRT, illustrating key terminology recommended by the Consolidated Standards of Reporting Trials (CONSORT) extension for SW-CRTs. One or more clusters are randomly allocated to each of several *sequences*, which dictate the order in which the intervention is implemented across participating clusters. The timing of implementation of the intervention is indicated by *steps*, with the number of steps and *step lengths* determined by design. Observations are taken repeatedly from each cluster in multiple *periods*. The numbers of clusters per sequence, sequences and periods, as well as average *cluster-period* sizes are key parameters required for sample size calculation for the SW-CRT.

The study diagram in Fig. 1 corresponds to that used in the RegisterNow-1 trial: a SW-CRT of a theory-based intervention to promote registration for organ and tissue donation in family physician waiting rooms in Ontario, Canada [10]. The intervention consisted of trained reception staff checking patients’ health cards for donor status and providing unregistered patients with a pamphlet that addresses barriers and facilitators to registration, in addition to Internet-enabled tablets to facilitate registration on the spot. Six clusters (family physician practices) were randomly allocated to one of six sequences. After an initial period in which all clusters were in the control condition, the intervention was implemented in one practice per step at intervals of 2 weeks for a total study duration of 14 weeks. Patients’ donor registration status at 7 days following their visit was obtained from provincial health administrative databases. The anticipated cluster-period size was 250 patient-visits for a total sample size of 10,500 patient visits.

## What are core methodological requirements for this design?

There are several fundamental requirements that are crucial to the successful execution of a SW-CRT. The first of these is to design the trial so the participants included in the trial are not selectively different between the intervention conditions under study. Such differences are broadly referred to as selection biases. The second requirement is to adopt an appropriate method of analysis so as to obtain an unbiased estimate of effectiveness of the intervention.

It is known that selection biases are more common in evaluations of policy interventions delivered at the level of the cluster and in trials which recruit participants after the treatment allocation is known [1]. There is reason to believe that these sorts of biases will also be more prevalent in the SW-CRT. This is because recruitment is likely to take place with knowledge of treatment allocation. One way to avoid these selection biases is to include data from every individual in the cluster, although this may be feasible only when routinely collected data are available and conditions for consent waivers are met.

Analysis of the SW-CRT is complicated by the fact that underlying changes over time – called secular trends – can influence the apparent effect of the intervention. In a SW-CRT, clusters gradually cross to the intervention so that data from those exposed to the intervention are obtained on average from a later calendar time. This means that mathematical modelling is needed to disentangle what are changes in outcomes due to secular trends and what are changes in outcomes due to the treatment being evaluated.

## What is the CONSORT extension for SW-CRTs?

The increased risks of biases associated with this design mean that SW-CRTs must be reported clearly to allow appropriate interpretation of their results. The Consolidated Standards Of Reporting Trials (CONSORT) Statement for individually randomised controlled trials provides guidance for reporting results from a randomised trial [12]. This guidance has been extended to cluster randomised trials and also to pragmatic trials [3, 13]. There is now a diverse range of reporting guidelines for randomised trials, and most are available on the EQUATOR website (http://www.equator-network.org/).

Several systematic reviews have demonstrated poor reporting of key methodological features of SW-CRTs; for this reason, an extension was developed for the SW-CRT [9]. The extension used EQUATOR-endorsed methodology and had full engagement with the EQUATOR Group throughout the process [11]. This statement has been produced with input from leading experts in stepped-wedge trial design who were invited to participate in a Delphi process to adapt the original CONSORT Statement. Final agreement for the proposed modifications was made at a 1-day consensus meeting. The consensus panel included journal editors, ethicists, statisticians, methodologists and developers of reporting guidelines.

## Highlights of issues for reporting

The crucial aspect of a SW-CRT that makes its reporting unique from the reporting of other trial designs is its longitudinal nature. These issues of temporality relate to the design, execution and reporting. The report of the study needs to carefully describe its design and this is often best depicted pictorially.

Of crucial importance is the partial confounding effect of calendar time and intervention exposure [8]. Few other randomised trials, by design, induce confounding. As stated earlier, time is a confounder in the SW-CRT because systematically more observations under the intervention condition are accrued later in calendar time than under the control condition. Authors must, therefore, report whether and how they adjusted for time in the analysis.

Other key aspects of reporting revolve around how participants (or their data), were recruited (or obtained) [4]. Where participants are continuously recruited into the trial, reporting whether this was done blind to the cluster allocation status is essential as it informs readers about the likely risks of recruitment biases. Where assessments on inclusion criteria were made with knowledge of the cluster allocation, the trial will be at increased risk of biases.

Of further importance is whether participants were repeatedly measured; or whether different participants were measured on each measurement occasion. Authors need to also describe how these repeated measures (on both participants and clusters) were accounted for in the sample size calculation and analysis [6]. In particular, conventional models used in the sample size formula and analysis of cluster randomised trials are unlikely to be valid in stepped-wedge trials, where correlations will depend not only on the cluster but also the time of measurement.

Other issues are whether there is any risk of within-cluster contamination (that is observations accrued under the control condition becoming contaminated with the intervention condition, and vice versa); whether there is any possibility that the effect of the intervention might vary over the duration of the study (perhaps because the intervention itself evolved); and justifications for the choice of a design (important for several reasons including the increase risk of bias and potentially exposing more clusters to the intervention) [9].

## How does the CONSORT Statement for SW-CRTs differ from the CONSORT Statement for cluster trials?

The CONSORT extension for SW-CRTs was developed primarily as an extension to the CONSORT Statement for individually randomised trials. However, the statement adapted the wording of the existing checklists (including the CONSORT Statement for cluster trials) and retained wording of other statements where possible. Whilst the SW-CRT is a cluster randomised trial, and so thus shares many reporting issues with the parallel-cluster trial, its longitudinal nature does make it different. Authors reporting SW-CRTs should follow the CONSORT Statement for SW-CRTs and do not need to additionally consult the SW-CRT for cluster trials. Also of relevance is appropriate reporting of any non-inferiority issues [15]; and pragmatic components [13]; a full description of the intervention [14]; and specific issues related to protocols or feasibility studies [2, 5]. In these cases, authors might need to ensure that they report according to multiple guidelines. A checklist of reporting items is available [9].

## Summary

Whilst the use of the SW-CRT is on the increase, there are still relatively few SW-CRTs being published; although its use is bound to show an exponential increase in the near future. By making reporting guidelines for this innovative design available relatively early in its development, it is our hope that the methodological conduct and reporting of future SW-CRTs will not be at the same risk of low quality of reporting as is the case with many other study designs.
